# Endovascular versus open surgery repair of intact abdominal aortic aneurysm: systematic review of randomized controlled trials and critical appraisal of meta-analyses

**DOI:** 10.1186/s13643-025-03044-2

**Published:** 2026-02-20

**Authors:** Frank Peinemann, Mohammed Bilal Olcay

**Affiliations:** 1 University Children’s Hospital of Cologne, Medical Faculty, University of Cologne, Kerpener Str. 62, Cologne, 50937 Germany; 2 FOM University of Applied Science for Economics & Management, Essen, Germany

**Keywords:** Aortic aneurysm, abdominal, Endovascular aneurysm repair, Angioplasty, Systematic review [publication type], Meta-analysis [publication type]

## Abstract

**Background:**

A few randomized controlled trials (RCTs) have investigated endovascular aneurysm repair (EVAR) and open surgical repair (OSR) in patients with intact aortic abdominal aneurysm (AAA).

**Objective:**

We aimed to evaluate the efficacy of EVAR vs OSR in patients with intact AAA.

**Methods:**

We included RCTs and searched MEDLINE and the Cochrane Library on 28 December 2024 without limits. The beneficial primary outcome was overall survival, and the adverse primary outcome was reintervention. In both cases, the outcome measure was the hazard ratio (HR). Two people independently selected studies and extracted data. Management of missing data included deducing time-to-event data from published survival curves and contacting study authors.

**Results:**

We retrieved 723 references from electronic databases and additional sources and included four RCTs. The pooled HR on overall survival favors neither EVAR nor OSR (HR = 1.02 [95% CI 0.93 to 1.12]), but the pooled HR on reintervention favors OSR (HR = 2.14 [95% CI 1.70 to 2.70]). Statistical heterogeneity was low, and the risk of bias had no noteworthy impact on the results.

**Conclusions:**

Pooled data from four randomized controlled trials on intact aortic abdominal aneurysm favor not EVAR regarding overall survival but favor OSR with respect to reintervention.

**Systematic review registration:**

PROSPERO CRD42024539841

**Supplementary Information:**

The online version contains supplementary material available at 10.1186/s13643-025-03044-2.

## Introduction

### Description of the condition

In this systematic review, the disease of interest is the intact abdominal aortic aneurysm. The diagnosis is usually established if the aortic diameter is 3.0 cm or larger [1]. Smoking is the strongest modifiable risk factor [2]. The natural course is characterized by increasing dilatation [3]. The aneurysm does not cause discomfort, though rupture is an imminent danger. Thus, early detection and repair are important to prevent the frequently lethal rupture.

### Description of the intervention

In this systematic review, the test intervention of interest is the endovascular aneurysm repair (EVAR). Open surgical repair (OSR) is the second treatment option and is the comparator in this systematic review. Patients with a diameter greater than 5.5 cm in men and 5.0 cm in women are eligible for either of the two repair procedures [4]. The aim of both interventions is to repair the aneurysm, thus preventing further expansion and rupture. EVAR is a type of minimally invasive endovascular surgery and involves the placement of an intraluminal stent graft using retrograde cannulation of the leg artery under local or regional anesthesia [5]. Open surgical repair (OSR) uses a surgical incision to access the aneurysm from the outside and replace the affected segment with a prosthetic graft.

### Objective

We aimed to assess the efficacy of endovascular aneurysm repair versus open surgical repair in patients with intact abdominal aortic aneurysm.

## Methods

While preparing this systematic review (SR), we endorsed the Preferred Reporting Items for Systematic Reviews and Meta-Analyses (PRISMA) statement [6] (see S1 Checklist for the complete questions, guidance, and checked PRISMA items). Patients and the public were not involved in this systematic review.

### Differences between protocol and review

The PROSPERO registration number is CRD42024539841. First, we classified overall survival as the beneficial primary outcome, and we classified reintervention as the adverse primary outcome. These two outcomes representing success and failure are both important to assess the overall benefit of the respective intervention. Second, we added the outcome aneurysm-related mortality as an additional secondary outcome to widen the scope of the reporting for subject-specific readers. Third, we added a critical appraisal of recently published systematic reviews on the condition that they reported on the same topic. Thereby, we extended the discussion of the context of our findings. We were interested not only in the results reported by the previously published systematic reviews but also in their methodological quality. Due to the comprehensiveness of the assessment, we inserted an additional section “critical appraisal of systematic reviews,” and we located this section before the discussion section.

### Inclusion criteria

We considered RCTs with parallel assignment of adult patients with an intact abdominal aortic aneurysm. The test intervention was endovascular aneurysm repair (EVAR) and the control intervention was open surgical repair (OSR).

We did not consider duplicate publications, abstract publications including meeting abstracts, ongoing studies, trial registry results, comments, letters, narrative reviews, study participants who were deemed unfit or ineligible for open repair (EVAR trial 2), surveillance versus EVAR, inflammatory abdominal aortic aneurysm, ruptured abdominal aortic aneurysm, predicting scores, nonrandomized studies, database analyses, systematic reviews, and meta-analyses. If costs or health-related quality of life were the only outcomes, then these studies were excluded.

Primary beneficial outcome was overall survival as a measure of the time from the beginning of the treatment until death. Synonymous terms include all-cause mortality, all-cause death, all deaths, death from any cause, or survival free of death. The corresponding measure of effect was the hazard ratio.

Primary adverse outcome was reinterventions as a measure of the time from the beginning of the treatment until the first treatment has failed and requires a second intervention afterwards. Synonymous terms include secondary (therapeutic) procedures. Reinterventions were defined as any surgical or endovascular procedures performed after the primary aneurysm repair procedure. The corresponding measure of effect was the hazard ratio.

We added aneurysm-related deaths as a secondary outcome. Aneurysm-related deaths were defined as the number of deaths related to the aneurysm or to the treatment. The corresponding measure of effect was the relative risk.

### Search strategy

We performed an unrestricted electronic literature database search in PubMed (U.S. National Library of Medicine) on 28 December 2024. Due to automated term mapping, use of an asterisk (*) as a wild card for truncation, different spelling, or use of synonyms was not necessary. We conducted an additional electronic literature database search in the Cochrane Central Register of Controlled Trials (CENTRAL) of The Cochrane Library via Wiley based on the PubMed search strategy. MeSH terms were combined with text terms to ensure a comprehensive and up-to-date search (see Table 1 for the search strategies).

**Table 1:** Search strategies

Source	Query	Retrieval	Search date
**1 MEDLINE** via PubMed using automated term mapping; no limits were used	Update search:	6	28 Dec 2024
	#6: #5 AND 2024/04/05:2024/12/28[edat]		
	Original search:	458	05 Apr 2024
	#1: random OR randomly OR randomized		
	#2: abdominal aortic aneurysm		
	#3: endovascular aneurysm repair		
	#4: open surgical repair		
	#5: #1 AND #2 AND #3 AND #4		
**2 Cochrane** Library via Wiley; no limits were used; 319 results: 264 Trials (CENTRAL) + 42 Reviews + 9 Protocols	Update search	3	28 Dec 2024
	#18: #17 with Cochrane Library publication date from Apr 2024 to Dec 2024, in Trials		
	Original search:	264	05 Apr 2024
	#: 1MeSH descriptor: [Random Allocation] explode all trees		
	#2: MeSH descriptor: [Randomized Controlled Trial] explode all trees		
	#3: MeSH descriptor: [Randomized Controlled Trials as Topic] explode all trees		
	#4: (random*):ti,ab,kw		
	#5: #1 OR #2 OR #3 OR #4		
	#6: MeSH descriptor: [Aortic Aneurysm, Abdominal] explode all trees		
	#7: (abdominal aortic aneurysm*)ti,ab,kw		
	#8: #6 OR #7		
	#9: MeSH descriptor: [Endovascular Aneurysm Repair] explode all trees		
	#10: (endovascular)ti,ab,kw		
	#11: (repair*)ti,ab,kw		
	#12: #10 AND #11		
	#13: #9 OR #12		
	#14: (open)ti,ab,kw		
	#15: (repair*)ti,ab,kw		
	#16: #14 AND #15		
	#17: #5 AND #8 AND #13 AND #16		

We searched in online trial registries on 28 December 2024 without applying limits. For searching in ClinicalTrials.gov (CT.gov, https://clinicaltrials.gov/, U.S. National Library of Medicine), we used the query “Endovascular aneurysm repair open surgical repair | Interventional studies.” For searching in the International Clinical Trials Registry Platform (ICTRP, https://trialsearch.who.int, World Health Organization), we used the query “Endovascular aneurysm repair open surgical repair.” We searched reference lists of recent publications, Google, and used PubMed tools including *similar articles* and *clinical queries*. We limited the search for meeting abstracts by relying on the pre-searched results retrieved from CENTRAL, which includes subject-related abstracts from Embase.

### Study selection

FP imported the bibliographic data into Excel (Microsoft) and EndNote X9 (Clarivate), and FP and MBO independently selected relevant studies in a two-step screening process. Differences in opinions were resolved by discussion, and the assistance of a third author was not necessary.

In the first step (title/abstract screening), we selected potentially relevant references by screening all imported references. We checked whether the title and/or abstract contained all expressions or concepts as follows: “endovascular” (aneurysm repair); “open” or conventional (surgical) repair; (unruptured or intact or elective) “abdominal aortic aneurysm”; “randomized” (controlled trial or comparison).

In the second step (full-text screening), we selected the references of studies to be included in this systematic review. We checked whether the full texts of the potentially relevant references met the inclusion criteria. The articles were required to be written in English or German. In addition, we screened the references to identify meta-analyses published in 2014 or later. We provided reasons for references excluded in the second step.

### Data extraction and analysis

FP and MBO independently extracted characteristics of the study design, participants, interventions, and outcomes into Word (Microsoft). Differences in opinions were resolved by discussion, and the assistance of a third author was not necessary. The main extracted data fields include the study characteristics as follows: registry number; study name; design; study locations; enrollment; inclusion, exclusion, and assignment criteria for study participants to enter; mean age and sex proportion of study participants; intervention arm; comparator arm; median follow-up; proportion of study participants lost to follow-up; outcomes relevant for this systematic review; time of the assessment of outcomes years from randomization.

Concerning the time-to-event data with respect to the reported Kaplan-Meier analyses on death from any cause as well as reintervention, we reenacted the survival functions by deducing survival data from the survival curves depicted in the corresponding figures of the articles. We used the Excel tool provided by Tierney 2007 to estimate the logHR (hazard ratio) and its standard error by using the “data from curve with numbers at risk given” [7], which is based on the method by Parmar 1998 [8]. We used the “data from curve read where wished and assuming constant censoring.” We made sure that the constructed graph based on inputted data was like the published graph. Data were analyzed using the Cochrane Review Manager 5 (The Cochrane Collaboration). We applied the statistical method *inverse variance*, the analysis model *random effects*, and the effect measure *hazard ratio*. We believe survival data representing the complete time from randomization to end of follow-up are more appropriate than selectively counting cumulative events at arbitrary time points.

Concerning dichotomous outcomes with respect to the reported incidences of aneurysm-related deaths, we extracted the number of patients in each treatment arm and the number of patients who experienced the outcome of interest. Data were analyzed using the Cochrane Review Manager 5 (The Cochrane Collaboration). We applied the statistical method *Mantel-Haenszel*, the analysis model *random effects*, and the effect measure *risk ratio*.

### Subgroups and heterogeneity

We assessed clinical reasons for heterogeneity and estimated the percentage heterogeneity between trials that cannot be ascribed to sampling variation using the index of heterogeneity (*I*^2^ statistic) [9]. An *I*^2^ statistic equal to or greater than 50% was regarded as substantial heterogeneity. The following sensitivity analyses were planned to explain at least parts of the statistical heterogeneity: studies with low risk of bias vs. high/unclear risk of bias and removal of studies with deviant data. Due to the low number of included studies and the lack of deviant data, those analyses were however not performed. Instead, we assessed the robustness of the results by comparing the random effects model versus the fixed effects model. Since we expected a small number of studies that enrolled patients within comparable time periods, we did not plan subgroup analyses.

### Risk of bias assessment

Two authors independently appraised the risk of bias of the included studies. MBO drafted an appraisal, and FP checked the draft against the text of the included articles. Differences in opinions were resolved by discussion, and the assistance of a third author was not necessary. We used the items listed within Cochrane’s tool for assessing risk of bias [10] and used seven items and amended criteria for judging an unclear, low, or high risk of bias (see Table 2 for a complete list of the risk of bias criteria). We also assessed the publication bias by visual inspection of the corresponding funnel plot [11].

**Table 2:** Risk of bias decision criteria

Item	Criteria for judging an unclear, low, or high risk of bias
Random sequence generation (selection bias)	Low: If adequately described Unclear: If not reported High: If not adequately described
Allocation concealment (selection bias)	Low: If adequately described Unclear: If not reported High: If not adequately described
Blinding of participants and personnel (performance bias)	Low: If blinding was addressed though not necessarily performed Unclear: If not reported High: If negated
Blinding of outcome assessment (detection bias)	Low: If adequately described Unclear: If not reported High: Not applicable because blinding of outcome assessment was regarded not appropriate
Incomplete outcome data (attrition bias)	Low: If the authors explained the excluded data and the attrition (lost other than death) was less than 10% in a single arm Unclear: If the attrition (lost other than death) was 10% or more but less than 20% in a single arm High: If the attrition (lost other than death) was 20% or more in a single arm
Selective reporting (reporting bias)	Low: If the study protocol is available, and the outcomes of interest have been reported in the pre-specified way Unclear: If a study protocol was not available and there was insufficient information to permit judgement High: If not all the pre-specified outcomes have been reported
Other bias	Low: If funding was not industry based Unclear: If funding and conflicts of interest were not reported High: If the sponsor provided industry-based funding and if authors were employees or consultants of the sponsor, or if the early discontinuation of the study was not predefined

## Results

### Search results

**Fig. 1:**
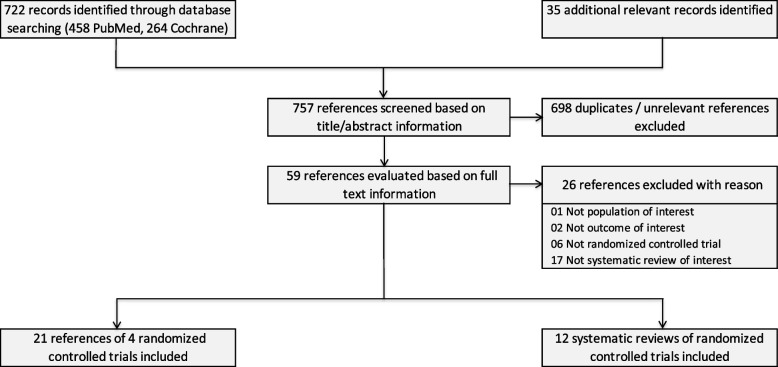
Flow diagram of the selection of studies

We retrieved 757 references from electronic databases and additional sources such as reference lists (see Fig. 1 for the flow diagram of the selection of studies). A total of 698 references were excluded based on title/abstract information. Another 26 references were read in full-text form but excluded from the review (see S2 Table for a full list of excluded references and corresponding exclusion reasons). Four RCTs (21 references) matched the inclusion criteria, and we included the articles reporting the most recent results, respectively [12–15]. For the supplemental evaluation of SRs, we included 12 systematic reviews of randomized controlled trials published since 2014 [16–27]. Thus, we included a total of 33 references (see S3 Table for a full list of included references).

### Study characteristics

The four included trials ACE, DREAM, EVAR1, and OVER [12–15] had industry-independent sponsors, enrolled study participants from 1999 to 2016 in 83 participating hospitals, and randomized 2,790 study participants (see Table 3 for an overview of the main characteristics of the included studies). All randomized data were analyzed based on the intention-to-treat principle. In most arms, 5% or fewer patients did not undergo the assigned intervention (range 1.1% to 8.3%). The studies included individuals with intact abdominal aortic aneurysm who were suitable candidates for either elective repair. Three studies requested an aortic diameter size of 5.0 cm, and one study used 5.5 cm. Study participants were mostly men (range 90.3% to 100% per arm), and the mean age was approximately 70 years. Median follow-up varied from three to about 12 years (range 0 to 15.8). The number of patients lost to follow-up was rather low, ranging from 0% to 3.3%.

**Table 3:** Study characteristics, overview

	**ACE (Becquemin 2011)**	**DREAM (van Schaik 2017)**	**EVAR1 (Patel 2016)**	**OVER (Lederle 2019)**
Registries	NCT00224718	NCT00421330	ISRCTN55703451	NCT00094575
Study name	ACE: Anevrysme de l'aorte abdominale, Chirurgie versus Endoprothese	DREAM: Dutch Randomised Endovascular Aneurysm Management	EVAR1: Endovascular aneurysm repair trial 1	OVER: Open Versus Endovascular Repair
Design	Randomized, parallel assignment, phase 3, no masking	Randomized, parallel assignment, phase 3, no masking	Randomized, parallel assignment, phase 3, no masking	Randomized, parallel assignment, phase 3, no masking
Sponsor	Assistance Publique—Hôpitaux de Paris, France	University Medical Center Utrecht, Netherlands	Health Technology Assessment Programme of the National Institute for Health Research, UK	Veterans Affairs Office of Research and Development, United States
Study locations	France: *N* = 25	Netherlands: *N* = 26; Belgium: *N* = 4	United Kingdom: *N* = 37	United States: *N* = 42
Enrollment	Mar 2003 to Mar 2008; number of screened patients not reported; 306 randomized; 299 analyzed	Nov 2000 to Dec 2003; number of screened patients not reported; 351 randomized and analyzed	Jul 1999 to Aug 2016; 4,799 screened; 1252 randomized and analyzed	Oct 2002 to Apr 2008; 5,162 screened; 881 randomized and analyzed
P: Inclusion	Abdominal aortic aneurysm at least 5.0 (4.5) cm in diameter in men (women); suitable candidates for either elective repair	Abdominal aortic aneurysm at least 5.0 cm in diameter; suitable candidates for either elective repair	Abdominal aortic aneurysm at least 5.5 cm in diameter; suitable candidates for either elective repair; at least 60 years of age	Abdominal aortic aneurysms at least 5.0 cm in diameter; suitable candidates for either elective repair
P: Assignment	2.6% (4 of 150) patients did not undergo assigned EVAR but completed OSR; 8.3% (18 of 149) patients did not undergo assigned OSR but 17 completed EVAR, 1 not any repair	1.1% (2 of 173) patients did not undergo assigned EVAR; 2.2% (4 of 178) patients did not undergo assigned OSR	2.5% (16 of 626) patients did not undergo assigned EVAR; 5.5% (35 of 626) patients did not undergo assigned OSR	3.8% (17 of 444) patients did not undergo assigned EVAR; 4.8% (21 of 437) patients did not undergo assigned OSR
P: mean age at randomization	EVAR: 68.9 years (SD 7.7); OSR: 70.0 years (SD 7.1)	EVAR: 70.7 years (SD 6.6); OSR: 69.6 years (SD 6.8)	EVAR: 74.1 years (SD 6.1); OSR: 74.0 years (SD 6.1)	EVAR: 69.6 years (SD 7.8); OSR: 70.5 years (SD 7.8)
P: male sex at randomization	EVAR: 100% (151 of 150); OSR: 97.9% (146 of 149)	EVAR: 93.1% (161 of 173); OSR: 90.4% (161 of 178)	EVAR: 90.3% (565 of 626); OSR: 91.1% (570 of 626)	EVAR: 99.3% (441 of 444); OSR: 99.5% (435 of 437)
Intervention arm	Endovascular repair (150 of 299)	Endovascular repair (173 of 351)	Endovascular repair (626 of 1252)	Endovascular repair (444 of 881 patients)
Comparator arm	Open surgical repair (149 of 299)	Open surgical repair (178 of 351)	Open surgical repair (626 of 1252)	Open surgical repair (437 of 881 patients)
Follow-up (years)	Median 3 years (range 0 to 4.8)	Median 10.2 years (quartile range 5.0 to 12.5)	Median 12.4 years (range 1.8 to 15.8)	Median 9.4 years (range 0.02 to 14.2)
Lost to follow-up	EVAR: 2% (3 of 150); OSR: 3.3% (3 of 149)	EVAR: 2.9% (5 of 173); OSR: 1.7% (3 of 178)	EVAR: 0% (0 of 626); OSR: 0.6% (4 of 626)	EVAR: 0% (0 of 444); OSR: 0% (0 of 437)
Outcome: prim; sec	Death from any cause; reintervention	Death from any cause; reintervention	Death from any cause; aneurysm-related deaths; reintervention	Death from any cause; reintervention

#### ACE

The authors of the study ACE [12] reported the combined outcome death from any cause or adverse events. Therefore, we sent an inquiry to the authors as to whether they could provide a follow-up analysis on long-term results that is planned or ongoing. We updated the email address and received a response that a follow-up analysis is not available. The authors kindly sent us separate Kaplan-Meier curves on death from any cause only and reintervention only, and we added the respective results.

#### DREAM

**Fig. 2:**
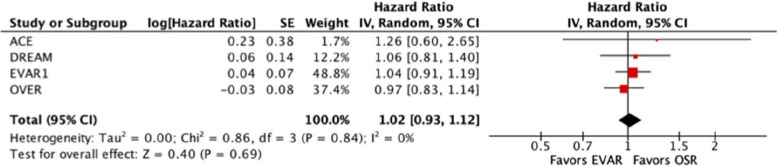
Forest plot EVAR versus OSR; event, death from any causes; measure, HR; 95% CI, 95% confidence interval; EVAR, endovascular aneurysm repair; HR, hazard ratio; *I*^2^, index of heterogeneity (the smaller the value, the lesser the heterogeneity); IV, inverse variance (statistical method); OSR, open surgical repair; P, probability; Random, random effects (analysis model); SE, standard error of log[Hazard Ratio]

The authors of the study DREAM [13] reported cumulative overall survival rates such as 38.5% for EVAR vs 42.2% for OSR at 12 years after randomization. Figure 2 of the article shows Kaplan-Meier curves, but the authors did not report the respective hazard ratio, 95% confidence interval, and Log-rank test. This information would not only be appropriate but expected. We used the curves to reenact the analysis and deduced the pertaining hazard ratio. In addition, the authors did not report alternative survival estimates such as odds ratio or relative risk. We used the number of events and participants of the two treatment arms and calculated the pertaining relative risk.

#### EVAR1

The authors of the study EVAR1 [14] reported Kaplan-Meier estimates for aneurysm-related survival up to 15 years of follow-up. The visual inspection obviously shows a crossing of the curves. Within the time interval from 6 to 8 years, they are superimposed upon each other. Before 6 years, the EVAR curve is above those of OSR, which suggests an advantage for EVAR in this earlier period. After 8 years, the EVAR curve is below those of OSR, which suggests an advantage for OSR in this later period. This curve crossing indicates a violation of the Kaplan-Meier assumptions. Thus, the hazard ratio and the p-value of the log-rank test representing the complete observation time are invalid.

#### OVER

The authors of the study OVER [15] did not report the Kaplan-Meier curve on the outcome reintervention. Therefore, we sent an inquiry to the authors whether they could provide the results. We received a response saying that the requested data are not available.

### Outcomes

**Fig. 3:**
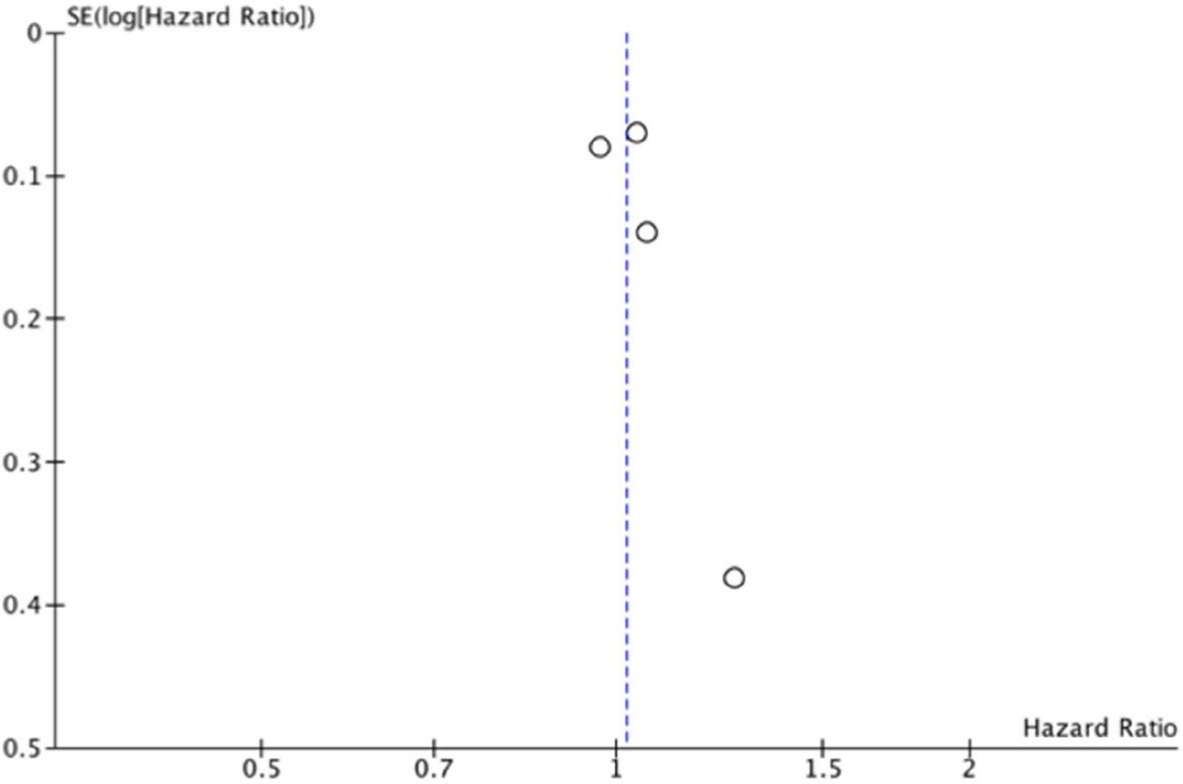
Overall mortality. Funnel plot pertaining to Fig. 2

The primary beneficial outcome overall survival was assessed by applying the hazard ratio as the effect measure on the event of death from any causes. The pooled hazard ratio favors no treatment groups, HR = 1.02 [95% confidence interval 0.93 to 1.12], the statistical heterogeneity is small, *I*^2^ = 0%, and visual inspection of the funnel plot does not suggest a relevant publication bias. See Table 4 for extracted and recalculated results and Fig. 2 for the forest plot and Fig. 3 for the funnel plot.

**Table 4:** Outcomes

	**ACE (Becquemin 2011)**	**DREAM (van Schaik 2017)**	**EVAR1 (Patel 2016)**	**OVER (Lederle 2019)**
***Death from all causes***				
HR extracted from paper	n.r	n.r	1.05, (95% CI 0.92 to 1.19)	0.96, (95% CI 0.82 to 1.13)
HR deduced from curve	1.26, (95% CI 0.60 to 2.65)	1.06, (95% CI 0.81 to 1.40)	1.04, (95% CI 0.91 to 1.19)	0.97, (95% CI 0.83 to 1.14)
Risk EVAR vs OSR extracted from paper	11% (17 of 150) vs 8% (12 of 149)	62% (106 of 173) vs 58% (103 of 178)	74% (466 of 626) vs 71% (444 of 626)	68.0% (302 of 444) vs 70.0% (306 of 437)
RR EVAR vs OSR calculated from risks	1.41, (95% CI 0.70 to 2.84)	1.06 (95% CI 0.89 to 1.26)	1.05 (95% CI 0.98 to 1.12)	0.97 (95% CI 0.89 to 1.06)
***Reintervention***				
HR extracted from paper	n.r	n.r	2.37, (95% CI 1.80 to 3.12)	n.r
HR dedu	3.03, (95% CI 1.21 to 7.62)	1.67, (95% CI 1.08 to 2.56)	2.29, (95% CI 1.78 to 2.96)	n.r
Risk EVAR vs OSR extracted from paper	16% (24 of 150) vs 2.68% (4 of 149)	38% (65 of 173) vs 21% (38 of 178)	26% (164 of 626) vs 12% (74 of 626)	26.7% (117 of 439) vs 19.8% (85 of 429)
RR EVAR vs OSR calculated from risks	5.96 (95% CI 2.12 to 16.76)	1.76 (95% CI 1.25 to 2.48)	2.22 (95% CI 1.72 to 2.85)	1.35 (95% CI 1.05 to 1.72)
***Aneurysm-related deaths***				
Risk EVAR vs OSR extracted from paper	4.0% (6 of 150) vs 0.7% (1 of 149)	4.7% (8 of 168) 7.4% (13 of 175)	9% (56/626) vs 7% (45/626)	2.7% (12 of 444) vs 3.7% (16 of 437)
RR EVAR vs OSR calculated from risks	5.96 (95% CI 0.73 to 48.91)	0.64 (95% CI 0.27 to 1.51)	1.24 (95% CI 0.85 to 1.81)	0.74 (95% CI 0.35 to 1.54)

*Abbreviations*: *CI *Confidence interval, *HR *Hazard ratio, *n.r. *Not reported, *RR *Risk ratio

**Fig. 4:**
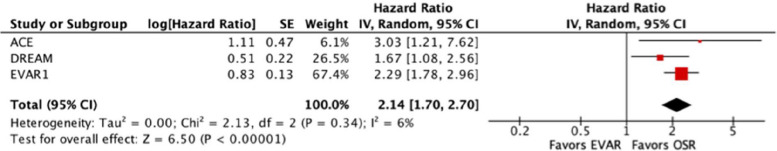
Forest plot EVAR versus OSR; event, reintervention; measure, HR; 95% CI, 95% confidence interval; EVAR, endovascular aneurysm repair; HR, hazard ratio; *I*^2^, index of heterogeneity (the smaller the value the lesser the heterogeneity); IV, inverse variance (statistical method); OSR, open surgical repair; P, probability; Random, random effects (analysis model); SE, standard error of log[Hazard Ratio]

The primary adverse outcome reintervention was also assessed by applying the hazard ratio as the effect measure on the event of any secondary (therapeutic) procedure. The pooled hazard ratio favors the open surgical repair group, HR = 2.14 [95% confidence interval 1.70 to 2.70], and the statistical heterogeneity was small, *I*^2^ = 6%. See Table 4 for extracted and recalculated results and Fig. 4 for the forest plot.

**Fig. 5:**
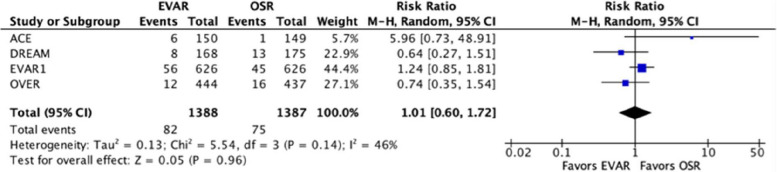
Risk of bias assessment, summary (left) and graph (right)

The added secondary outcome aneurysm-related deaths was assessed by applying the risk ratio as the effect measure because the hazard ratio was not available. Three studies [12–15] did not report the respective survival curves and one study [14], as mentioned above, reported invalid data. The pooled risk ratio favors no treatment group, RR = 1.01 [95% confidence interval 0.60 to 1.72], and the statistical heterogeneity was moderate, *I*^2^ = 46%. See Table 4 for extracted and recalculated results and Fig. 5 for the forest plot. Removal of the ACE study data resulted in a noteworthy reduction in *I*^2^ of 32%. The ACE study had to stand a follow-up that was considerably shorter than those of the other three studies. The shorter time for the observation of events could have contributed to a low number of events and a remarkably wide confidence interval. This can in part explain the heterogeneity. Transition from the random effects model to the fixed effects model did not result in a noteworthy impact.

### Results of the risk of bias assessment

**Fig. 6:**
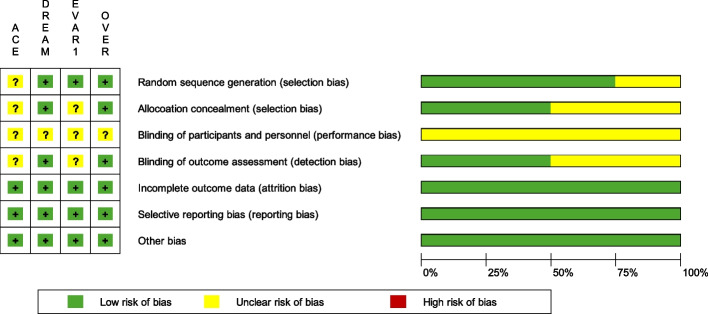
AMSTAR 2 assessment: summary (left) and graph (right). A1, AlOthman 2020; A2, Antoniou 2020; B, Bulder 2019; C, Chen 2019; G, Giannopoulos 2020; K, Kontopodis 2023; L1, Li 2019; L2, Loufopoulos 2023; P, Paravastu 2014; S, Shi 2020; T, Takagi 2017; W, Wang 2023; Y, Yokoyama 2020. Count +/p/-, number of Yes/partial yes/NO for the respective questions. *Percent +/p/-*, percentage of Yes/partial yes/NO for the respective questions. The Fig. 12 stands for the total number of the critically appraised systematic reviews

We conducted 28 assessments per reviewer, seven items for any of the four included RCTs. MBO drafted and explanations, and FP cross-checked all items (see Table 5 for verbalized results and Fig. 6 for summary and graph of the risk of bias assessment). Each assessment contains a statement whether a low, unclear, or high bias was judged and adds a reasonable explanation for the respective decision. About two-thirds were judged low risk, and about one-third was judged unclear bias. Thus, there was no judgement of a high risk of bias in any of the items. The item Blinding of participants and personnel (performance bias) received an unclear risk of bias judgement across all RCTs adding up to almost half of all the unclear bias judgements. All four RCTs received funding independent from industry.

**Table 5:** Results of the risk of bias assessment

Item	ACE (Becquemin 2011)	DREAM (van Schaik 2017)	EVAR1 (Patel 2016)	OVER (Lederle 2019)
Random sequence generation (selection bias)	Becquemin 2009 (Protocol): “Data were collected by fax from the various centers at the Clinical Research Unit (URC) of Henri Mondor Hospital. After randomization by the center, arm allocation was notified 24 h.” Becquemin 2011: “Patient data in each center were checked by a multidisciplinary team (vascular surgeon, radiologist, and anesthesiologist). After written informed consent was obtained, the Clinical Research Unit of Henri Mondor Hospital performed randomization stratified by center. Arm allocation was notified 24 h to the investigator.” The actual random sequence generation was not described. Thus, we judged an unclear risk of bias	de Bruin 2010: “Randomization to either procedure was carried out centrally with the use of a computer-generated, permuted-block sequence and stratified according to study center in blocks of four patients.” The random sequence generation was adequately described. Thus, we judged a low risk of bias	Greenhalgh 2010: “Randomisation was performed using a 1:1 ratio in randomly permuted blocks and varying block sizes constructed using the Stata software package. Randomisation was stratified by centre such that local differences in decisions on anatomical suitability and fitness for open repair would not lead to differences between randomised groups. Randomisation was performed by the trial manager once all baseline data had been received at the central trial management office.” Thus, we judged a low risk of bias	Lederle 2012: “Patients were randomly assigned to one of the two repair procedures in a 1:1 ratio.” We assume that the involved coordinating center provided for an adequate random sequence generation. Thus, we judged a low risk of bias
Allocation concealment (selection bias)	The actual random sequence generation was not described. Thus, we judged an unclear risk of bias	Prinssen 2002. “The surgeon then contacts the randomisation center by telephone and the result of the randomisation is given immediately.” The allocation concealment was adequately described. Thus, we judged a low risk of bias	Allocation concealment not reported. Thus, we judged an unclear risk of bias	Lederle 2012: “Though group assignments were of necessity unblinded, concealment of treatment as assignments from site investigators and patients was maintained throughout randomization by means of telephone calls to the coordinating center.” We assume that the involved coordinating center provided for an adequate allocation concealment. Thus, we judged a low risk of bias
Blinding of participants and personnel (performance bias)	Blinding of participants and personnel was not reported. Thus, we judged an unclear risk of bias	Blinding of participants and personnel was not reported. Thus, we judged an unclear risk of bias	Blinding of participants and personnel was not reported. Thus, we judged an unclear risk of bias	Lederle 2009: “Although patient assignment was of necessity unblinded, outcome data by treatment group were available during enrollment only to the biostatistician and data monitoring committee.” Thus, we judged an unclear risk of bias
Blinding of outcome assessment (detection bias)	Blinding of outcome assessment was not reported. Thus, we judged an unclear risk of bias	de Bruin 2010: “The long-term primary outcomes were rates of death from any cause and reintervention. An out- come-adjudication committee, consisting of five vascular surgeons, classified the causes of death and reinterventions in a blinded fashion and in- dependently from one another. Disagreements were resolved in a plenary consensus meeting.” Blinding of outcome assessment was applied. Thus, we judged a low risk of bias	Blinding of outcome assessment was not reported. Thus, we judged an unclear risk of bias	Lederle 2009: “Although patient assignment was of necessity unblinded, outcome data by treatment group were available during enrollment only to the biostatistician and data monitoring committee.” Thus, we judged a low risk of bias
Incomplete outcome data (attrition bias)	In the EVAR arm 2% were lost to follow-up and in the OSR 3.3%. Thus, we judged a low risk of bias	de Bruin 2010: “Survival was calculated with the use of Kaplan–Meier methods on an intention-to-treat basis. […] Not a single patient was lost to follow-up, and all surviving patients were followed for at least 5 years after randomization.” Thus, we judged a low risk of bias	Greenhalgh 2010: “All analyses were performed according to a pre-defined statistical-analysis plan and were based on the intention-to-treat principle. [..] The median follow-up until death or the end of the study was 6.0 years (interquartile range, 3.9 to 7.3), and only 1% of patients were lost to follow-up in terms of mortality.” Thus, we judged a low risk of bias	Lederle 2009 reported that two were lost to follow-up in the EVAR arm and non in the OSR arm. The analysis was by intention-to-treat. Thus, we judged a low risk of bias
Selective reporting (reporting bias)	Patient flow and primary/secondary outcomes were provided, and treatment groups were evenly balanced for baseline characteristics and an intention-to-treat analysis was applied. The study protocol is available, and the outcomes of interest have been reported in the pre-specified way. Thus, we judged a low risk of bias	The study protocol is available, and the outcomes of interest have been reported in the pre-specified way. Thus, we judged a low risk of bias	The study protocol is available, and the outcomes of interest have been reported in the pre-specified way. Thus, we judged a low risk of bias	The study protocol is available, and the outcomes of interest have been reported in the pre-specified way. Thus, we judged a low risk of bias
Other bias	NCT00224718: “The sponsor: Assistance Publique—Hôpitaux de Paris.” Funding was not industry based. Thus, we judged a low risk of bias	NCT00421330: “Sponsor: UMC Utrecht”. Funding was not industry based. Thus, we judged a low risk of bias	Greenhalgh 2010: “The trial was sponsored by the Health Technology Assessment Programme of the National Institute for Health Research in the United Kingdom. No support was provided by pharmaceutical or medical-device companies.” Thus, we judged a low risk of bias	Lederle 2009: “This work was supported by the Co-operative Studies Program of the Department of Veterans Affairs (VA) Office of Research and Development, Washington, DC.” Thus, we judged a low risk of bias

### Critical appraisal of systematic reviews

#### Inclusion criteria

We considered systematic reviews (SRs) on the condition that these SRs had reported pooled effects as part of a quantitative assessment of RCTs on the outcome overall survival on randomized controlled trials on EVAR versus OSR and were published from 2014 onwards. We resorted to the original retrieval and did not conduct a separate search and included systematic reviews (SRs).

#### Search results

For the supplemental evaluation of SRs, we included 12 systematic reviews [16–27] (see Fig. 1 for the flow diagram of the selection of studies).

#### Data extraction and analysis

FP and MBO independently extracted characteristics of the SRs into Word (Microsoft). We extracted search dates and numbers of included RCTs and NRSs in the study as well as in the meta-analysis, and we extracted the findings with respect to overall survival. We extracted the hazard ratio (HR) if reported; otherwise, we extracted alternative effect measures such as the odds ratio (OR). We listed only one overall mortality estimate per systematic review, and we chose the longest reported follow-up time, e.g., any time over late over intermediate over early mortality. The *I*^2^ (I squared) describes the percentage of the variability in effect estimates that is due to heterogeneity; the smaller the value, the lesser the heterogeneity. If not reported, we calculated I squared according to the following formula: I squared = ((chi-squared statistic minus degrees of freedom)/chi-squared statistic) × 100%.

#### Critical appraisal approach

Two authors independently appraised the quality of the included systematic reviews. MBO and FP prepared separate judgement sheets, and we conducted a consensus discussion to resolve differences in opinions. We adopted AMSTAR 2 [28], a critical appraisal tool for SRs that includes 16 questions as well as guiding notes when to answer Yes, Partial yes, or NO (see Table 6 for a complete list of the amended AMSTAR 2 items). Concerning question "4”, we did not require the inclusion/consulting of a content experts in the field and the searching for grey literature. It should be noted that AMSTAR 2 is not designed to generate an overall score. Jüni [29] reported, quote: “that the use of summary scores to identify trials of high quality is problematic. Relevant methodological aspects should be assessed individually and their influence on effect sizes explored.” We went beyond AMSTAR 2 to detect various other methodological issues and ascribed those to the respective article.

**Table 6:** AMSTAR 2 assessment: 16 items

Questions and guidance for answering yes and/or partial yes
1. Did the research questions and inclusion criteria for the review include the components of PICO? For Yes: PICO
2. Did the report of the review contain an explicit statement that the review methods were established prior to the conduct of the review and did the report justify any significant deviations from the protocol? For Partial Yes: Written protocol that included review question(s) AND search strategy AND inclusion/exclusion criteria AND risk of bias assessment For Yes: AND protocol should be registered AND meta-analysis/synthesis plan AND plan for investigating causes of heterogeneity
3. Did the review authors explain their selection of the study designs for inclusion in the review? For Yes: Explanation for including only RCTs OR both RCT and NRS
4. Did the review authors use a comprehensive literature search strategy?^(a)^ For Partial Yes: Searched at least 2 databases AND provided key word and/or search strategy AND justified publication restrictions (e.g. language) For Yes: AND searched the reference lists/bibliographies of included studies AND searched trial/study registries AND conducted search within 24 months of completion of the review
5. Did the review authors perform study selection in duplicate?
6. Did the review authors perform data extraction in duplicate?
7. Did the review authors provide a list of excluded studies and justify the exclusions? For Partial Yes: Provided a list For Yes: AND justified the exclusion from the review of each potentially relevant study
8. Did the review authors describe the included studies in adequate detail? For Partial Yes: PICO AND research design For Yes: AND study's setting AND timeframe for follow-up
9. Did the review authors use a satisfactory technique for assessing the risk of bias (RoB) in individual studies that were included in the review? For Partial Yes (RCT): Unconcealed allocation AND lack of blinding of patients and assessors when assessing outcomes (unnecessary for objective outcomes such as all-cause mortality) For Yes (RCT): AND allocation sequence that was not truly random, and selection of the reported result from among multiple measurements or analyses of a specified outcome
10. Did the review authors report on the sources of funding for the studies included in the review?
11. If meta-analysis was performed did the review authors use appropriate methods for statistical combination of results? For Yes: justified combining the data in a meta-analysis AND used an appropriate weighted technique AND investigated the causes of any heterogeneity
12. If meta-analysis was performed, did the review authors assess the potential impact of RoB in individual studies on the results of the meta-analysis or other evidence synthesis? For Yes: Performed analyses to investigate possible impact of RoB on summary estimates of effect
13. Did the review authors account for RoB in individual studies when interpreting/discussing the results of the review? For Yes: If RCTs with moderate or high RoB, or NRS were included the review provided a discussion of the likely impact of RoB on the results
14. Did the review authors provide a satisfactory explanation for, and discussion of, any heterogeneity observed in the results of the review? For Yes: No significant heterogeneity OR investigation of sources of any heterogeneity and discussion of the impact of this on the results
15. If they performed quantitative synthesis did the review authors carry out an adequate investigation of publication bias (small study bias) and discuss its likely impact on the results of the review?
16. Did the review authors report any potential sources of conflict of interest, including any funding they received for conducting the review?

Abbreviations: *NR*S, nonrandomized studies; *PICO*, population, intervention, comparator, outcome; *RCT*, randomized controlled trial; *RoB*, risk of bias

^(a)^ Question 4. We removed the following criteria because they were deemed not relevant: “AND included/consulted content experts in the field AND where relevant, searched for grey literature”

#### Characteristics of systematic reviews

Search dates of the 12 included SRs range from Dec 2012 to Jan 2023 [16–27]. Only three SRs relied solely on evaluating RCTs [17, 20, 24] and nine SRs included NRSs for evaluating the effect of EVAR vs OSR (see Table 7 for the individual studies included in any of the 12 SRs). The four RCTs (ACE, DREAM, EVAR1, and OVER) of this work were also included in eight of the 12 SRs. Five SRs [17, 20, 23–25] included five additional RCTs [30–34] that did not meet the inclusion criteria of the present work. Chen [30] investigated mortality at 3-month follow-up. Cuypers [31] investigated cardiac responses using electrocardiography, echocardiography, clinical observation, and cardiac enzymes but not mortality. EVAR2 [32] investigated endovascular repair of aortic aneurysm in patients physically ineligible for open surgical repair. Lottman [33] investigated health-related quality of life scores such as SF-36 but not mortality. We expressed reservations about Soulez [34] because the curve crossing of the overall mortality time-to-event analysis indicated a violation of the Kaplan-Meier assumptions.

**Table 7:** Included systematic reviews

SR IDs	Search dates	ACE	DREAM	EVAR1	OVER	Chen 2011	Cuypers 2001	EVAR2	Lottman 2004	Soulez 2005	NRS
*Present analysis*	*28 Dec 2024*	*Yes*	*Yes*	*Yes*	*Yes*	*–*	*–*	*–*	*–*	*–*	*–*
AlOthman 2020 [16]	–	Yes	Yes	Yes	Yes	–	–	–	–	–	6
Antoniou 2020 [17]	Jul 2019	Yes	Yes	Yes	Yes	Yes	–	–	Yes	Yes	–
Bulder 2019 [18]	17 Mar 2018	Yes	Yes	Yes	Yes	–	–	–	–	–	49
Chen 2019 [19]	Nov 2017	Yes	Yes	Yes	Yes	–	–	–	–	–	7
Giannopoulos 2020 [20]	Jun 2019	Yes	Yes	Yes	Yes	–	–	–	–	Yes	–
Kontopodis 2023 [21]	30 Mar 2022	–	–	–	Yes	–	–	–	–	–	15
Li 2019 [22]	May 2018	Yes	Yes	Yes	–	–	–	–	–	–	68
Loufopoulos 2023 [23]	14 Jan 2023	Yes	Yes	Yes	Yes	–	–	–	–	Yes	8
Paravastu 2014 [24]	Dec 2012	Yes	Yes	Yes	Yes	–	–	Yes	–	–	–
Shi 2020 [25]	Dec 2018	–	Yes	Yes	–	–	Yes	–	–	Yes	34
Takagi 2017 [26]	Aug 2016	–	Yes	Yes	–	–	–	–	–	–	5
Yokoyama 2020 [27]	Nov 2019	Yes	Yes	Yes	Yes	–	–	–	–	–	7

*Abbreviations*: – not reported/not included, *NRS *Nonrandomized studies

#### Critical appraisal results

**Fig. 7:**
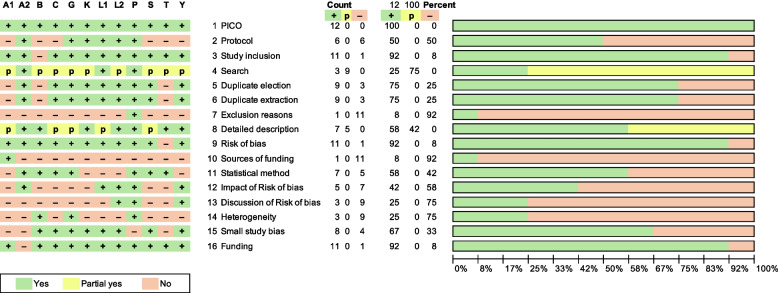
AMSTAR 2 assessment: summary (left) and graph (right). A1, AlOthman 2020 [16]; A2, Antoniou 2020; B, Bulder 2019; C, Chen 2019; G, Giannopoulos 2020; K, Kontopodis 2023; L1, Li 2019; L2, Loufopoulos 2023; P, Paravastu 2014; S, Shi 2020; T, Takagi 2017; W, Wang 2023; Y, Yokoyama 2020. Count +/p/-, number of Yes/partial yes/NO for the respective questions. *Percent +/p/-*, percentage of Yes/partial yes/NO for the respective questions. The Fig. 12 stands for the total number of the critically appraised systematic reviews

Of the 12 included SRs, eight SRs reported the HR and four reported the OR as the summary effect measure for overall mortality (see Table 8 for an overview of the respective meta-analyses estimates and contributing studies). The second and the third columns from the left show whether the resulting estimates of the meta-analyses were calculated from data of RCTs, NRSs, or both. Six SRs summarized only RCT data [17–20, 23, 24], and the statistical heterogeneity was low. The *I*^2^ equaled 0% with one exception. The other six SRs [16, 21, 22, 25–27] put RCTs and NRSs data together, and statistical heterogeneity was significant. The *I*^2^ ranged from 34% to 89% with one exception. The summary estimate of overall mortality favors not any treatment alternative in 11 SRs. One SR [26] conducted a split time-point analysis and reported solely the result of the late time interval. Thus, the results of this SR can hardly be compared to the other SRs. While reading the papers, we detected potential methodological issues (see Table 9 for a complete list of methodological issues for each SR). For example, data from up to seven RCTs and 68 NRSs were lumped together, Kaplan-Meier assumptions were violated but not adequately considered, the odds ratio was used instead of relative risk or hazard ratio, and outdated data were used instead of up-to-date data. The AMSTAR 2 required the assessment of 16 individual items to critically appraise 12 SRs per reviewer (see Table 10 for the verbalized AMSTAR 2 results and Fig. 7 for summary and graph). Before we reached a consensus about differences in opinion, the Cohen’s kappa coefficient was 0.69, which was quite acceptable (see Table 11 for the calculation of Kappa). Item four (search strategy) was frequently judged “partially yes” because the search in reference lists was not reported. The provision of a list of references that were initially regarded as potentially relevant and the respective exclusion reason (question 7) was only reported by the Cochrane Review; thus, the answer was “NO” for all other SRs. There were two questions on industry funding. Question 10 relates to the authors of the RCTs, and the funding was not reported in 11 SRs. Question 16 relates to the authors of the SRs, and the funding was not reported in one SR. 11 SRs reported results on the risk of bias assessment. Frequently, we did not find a statement on the assessment and discussion of the impact of the risk of bias results on the summary estimates of effect. We found that the response to both relating questions 12 and 13 was not an easy task.

**Table 8:** Effect measures reported in systematic reviews

SR IDs	RCT	NRS	HR [95% CI]	OR [95% CI]	I^2^	Location
*Present analysis*	*4*	*0*	*1.02 [0.93, 1.12]*	*–*	*0%*	Figure 2
AlOthman 2020 [16]	3	4	–	0.98 [0.75, 1.29]	87%	Figure 2, analysis 1.2.4
Antoniou 2020 [17]	3	–	1.02 [0.93, 1.13]	–	0%	Figure 4A
Bulder 2019 [18]	3	–	1.00 [0.91, 1.10]	–	0%	Figure 2
Chen 2019 [19]	4	–	–	0.90 [0.76, 1,06]	0%	Figure 5
Giannopoulos 2020 [20]	4	–	1.04 [0.93, 1.17]	–	16%	Figure 2
Kontopodis 2023 [21]	1	7	1.38 [0.81, 2.33]	–	76%	Figure 2B
Li 2019 [22]	2	13	–	0.92 [0.76, 1.11]	89%	Figure 3
Loufopoulos 2023 [23]	5	–	0.97 [0.88, 1.07]	–	0%	Supplement Fig. 26
Paravastu 2014 [24]	3	–	–	0.98 [0.83, 1.15]	0%	Analysis 1.3
Shi 2020 [25]	3	14	1.03 [0.96, 1.10]	–	0%	Figure 7
Takagi 2017 [26]	2	5	1.23 [1.03, 1.47]	–	60%	Figure 2 (only late phase)
Yokoyama 2020 [27]	3	3	1.17 [0.93, 1.47]	–	34%	Figure 3D

*Abbreviations*: – not reported/not included, *CI* Confidence interval, *HR* Hazard ratio, *I*^2^ Index of heterogeneity, *NRS* Nonrandomized studies, *OR* Odds ratio

**Table 9:** Detected issues in systematic reviews

ID	Comments
AlOthman 2020 [16]	Issue 1: The authors used only the odds ratio, but they should have used the relative risk to prevent misleading [35]. There are reasons to use odds ratios only in case-control studies and logistic regression analyses [36] Issue 2: The authors reported only the number of events and participants but not time-to-event data nor Kaplan-Meier curves Issue 3: According to Fig. 2 (analysis 1.2.4), EVAR vs OSR yielded 576 vs 589 events in 1066 vs 920 participants. The resulting odds ratio is 0.66 [0.55, 0.79] but the resulting relative risk would be 0.84 [0.78, 0.91]; 95% confidence interval are shown in square brackets Issue 4: The authors reported subtotals of four different meta-analyses on all-cause mortality that differed by follow-up time (30 days or during hospital stay; two years; four years; six years or longer), studies, and design. Notably, randomized and nonrandomized data were lumped together. Then, a total was calculated by adding all figures of those four analyses, thereby producing duplicate data
Antoniou 2020 [17]	Issue 1: The authors included 7 randomized trials (ACE, Chen 2011, DREAM, EVAR1, Lottman 2004, OVER, Soulez 2005), though used only 3 studies (EVAR1, DREAM, OVER) in the meta-analysis on all-cause mortality any time Issue 2: Trials were excluded if judged to be of high risk of bias in two or more domains. Usually, the complete number of RCTs should be included. Then, the robustness is tested in a sensitivity analysis comparing low risk vs. unclear risk or high risk of bias Issue 3: The authors reported that studies with short or medium term follow up (< 8 years) were excluded from time-to-event analyses. The analysis uses the Kaplan-Meier method. According to D'Arrigo 2021 [37], quote: “the Kaplan-Meier method estimates the conditional probability of survival calculated at specific time points dictated by the occurrence of the event.” Censoring incomplete observations is a characteristic of the Kaplan-Meier method, and it is not necessary that participants or studies need identical observation times. Therefore, we think that it is not compulsory to exclude studies based on various follow-up times
Bulder 2019 [18]	Issue 1: The authors conducted three meta-analyses on overall survival and calculated subtotals. These analyses differ by follow-up and studies Issue 2: In each of three meta-analyses on overall survival, randomized studies, administrative registry studies, and cohort studies were analyzed separately. These analyses differ by follow-up. Within each meta-analysis, data of all included studies of various designs were lumped together and used to calculate a total
Chen 2019 [19]	Issue 1: The authors used only the odds ratio, but they should have used the relative risk to prevent misleading [35]. There are reasons to use odds ratios only in case-control studies and logistic regression analyses [36] Issue 2: The authors reported only the number of events and participants but not time-to-event data nor Kaplan-Meier curves Issue 3: The authors reported subtotals of two different meta-analyses on all-cause mortality resulting in a subtotal for randomized and a subtotal for nonrandomized studies. Then, a total was calculated by adding all figures of those two analyses, thereby mixing randomized and nonrandomized data
Giannopoulos 2020 [20]	Issue 1: The authors conducted three meta-analyses on mortality (all-cause; follow-up at 4 years to 8 years; follow-up at 8 years and more) and included the same three studies in all those analyses, though they did not calculate a total Issue 2: In the abstract, the authors stated, quote: “Hazard ratios were calculated for mortality at different time intervals”. According to D'Arrigo 2021 [37], quote: “the Kaplan-Meier method estimates the conditional probability of survival calculated at specific time points dictated by the occurrence of the event.” We think that all available information should be used in a time-to-event analysis. Thus, excluding studies or data based on a shorter follow-up might not be helpful. If there is a substantial crossing in the course of the Kaplan-Meier curves, then the crossing point could be used as a division point the distinguishes between the early and the late events. In this case, the crossing acts as the end point of the early time interval and as the start point of the late time interval Issue 3: In the abstract, the authors stated, quote: “risk ratios were calculated in cases where the total number of events was available.” If there are time-to-event data available, which is the case, then risk ratios may be of minor importance or possibly redundant
Kontopodis 2023 [21]	Issue 1: The authors included only participants < 70 years of age. This age cut-off appears arbitary. The median age at diagnosis was reported 69 years for men and 78 years for women [38]. Li 2019 included 299,784 patients in a meta-analysis on EVAR vs OSR, and the mean age of patients ranged from 56 to 84 years Issue 2: The OVER study reported various subgroups on hazard ratios for death. The < 70 years of age subgroup contained 406 participants and the > = 70 years of age subgroup contained 475 participants. The respective data were extracted into the meta-analysis. Because of that, a single RCT was included into the meta-analysis beside 15 observational studies. In Fig. 2 (B), the authors calculated a total hazard ratio on overall mortality. This meta-analysis pools the data of the randomized study with those of seven observational studies. We think that this is not appropriate. Furthermore, one of the observational study in the same meta-analysis shows a hazard ratio of more than 200. Both issues questions the credibility of the work
Li 2019 [22]	Issue 1: The authors used only the odds ratio, but they should have used the relative risk to prevent misleading [35]. There are reasons to use odds ratios only in case-control studies and logistic regression analyses [36] Issue 2: The authors reported only the number of events and participants but not time-to-event data nor Kaplan-Meier curves Issue 3: The authors reported two different meta-analyses on all-cause mortality that differed by follow-up time (5 years or longer; 10 years or longer) Issue 4: Those two meta-analyses combine the data of the three randomized studies with those of 68 observational studies. We think that this is not appropriate Issue 5: Concerning the three included randomized studies, the authors used older articles (DREAM 2010, EVAR1 2010, OVER 2012) for the shorter follow-up meta-analysis and newer articles (DREAM 2017, EVAR1 2016) for the longer follow-up meta-analysis. We think that this is irregular Issue 6: The I square of both meta-analyses on all-cause mortality is about 90% representing a considerable statistical heterogeneity. The forest plot may be used to show the inconsistent results among individual studies. However, the calculation of a pooled estimate should be omitted to avoid biased interpretations
Loufopoulos 2023 [23]	Issue 1: Fig. 1 shows two Kaplan-Meier curves representing overall survival of EVAR vs OSR. Visual inspection suggests that the proportional hazards assumption is violated. The authors realized this and conducted a split time-point analysis. In general, the time point of the crossing of the curves is used to separate the time course in an early and a late time period resulting in two separate survival analyses. Nevertheless, the authors reported the invalid hazard ratio of the complete and undivided analysis in the abstract, which should not be done Issue 2: The pooled Kaplan-Meier analysis shown in Fig. 1 lumps together the data of the five included randomized studies with those of eight nonrandomized studies. We think that this is not appropriate Issue 3: The title announces long-term outcomes. However, the authors included the outdated DREAM 2010 instead of the newer van DREAM 2017 and the outdated EVAR1 2010 instead of the newer EVAR1 2016, respectively Issue 4: The authors included the randomized study of Soulez 2005. Concerning the results on overall mortality, the curve crossing indicates a violation of the Kaplan-Meier assumptions
Paravastu 2014 [24]	Issue 1: The authors used only the odds ratio, but they should have used the relative risk to prevent misleading [35]. There are reasons to use odds ratios only in case-control studies and logistic regression analyses [36] Issue 2: The authors reported only the number of events and participants but not time-to-event data nor Kaplan-Meier curves
Shi 2020 [25]	Info: The authors included four RCTs (Cuypers, DREAM, EVAR1, Soulez) and 55 cohort studies Issue 1: The meta-analysis on all-cause mortality (Fig. 7) combines the data of three randomized studies (DREAM, EVAR1, Soulez 2005) with those of 14 nonrandomized studies. We think that this is not appropriate
Takagi 2017 [26]	Info: The authors included two RCTs (DREAM, EVAR1) and seven propensity-score matching studies Issue 1: The authors excluded OVER because the respective publication in 2009 did not meet the inclusion criteria of the follow-up time of at least 5 years. We think that the authors could have included OVER because an updated publication in 2012 reported a follow-up of nine years Issue 2: Fig. 1 shows two Kaplan-Meier curves representing overall survival of EVAR vs OSR. Visual inspection suggests that the proportional hazards assumption is violated. The authors realized this and conducted a split time-point analysis. In general, the time point of the crossing of the curves is used to separate the time course in an early and a late time period resulting in two separate survival analyses. The authors reported only the late phase between 1.8 and 5.0 years but not the early phase between 0 and 1.8 years Issue 3: The HR of the late phase was given 1.29 [1.24, 1.35] (I^2^: not reported) in Fig. 1 but 1.23 [1.03, 1.47] (*I*^2^ = 60%) in Fig. 2
Yokoyama 2020 [27]	Info: The authors included four RCTs (ACE, DREAM, EVAR1, OVER) and seven propensity-score matching studies Issue 1: The pooled Kaplan-Meier analysis shown in Fig. 3 lumps together the data of the randomized studies with those of the nonrandomized studies. We think that this is not appropriate

*Abbreviations*: *RCT* Randomized controlled trial

**Table 10:** Results of AMSTAR 2 assessment

#	AlOthman 2020 [16]	Antoniou 2020 [17]	Bulder 2019 [18]	Chen 2019 [19]	Giannopoulos 2020 [20]	Kontopodis 2023 [21]	Li 2019 [22]	Loufopoulos 2023 [23]	Paravastu 2014 [24]	Shi 2020 [25]	Takagi 2017 [26]	Yokoyama 2020 [27]
1	Yes	Yes	Yes	Yes	Yes	Yes	Yes	Yes	Yes	Yes	Yes	Yes
2	NO	Yes	NO	NO	Yes	Yes	Yes	Yes	Yes	NO	NO	NO
3	Yes	Yes	NO	Yes	Yes	Yes	Yes	Yes	Yes	Yes	Yes	Yes
4	(yes)	Yes	(yes)	(yes)	(yes)	(yes)	Yes	(yes)	Yes	(yes)	(yes)	(yes)
5	NO	Yes	NO	Yes	Yes	Yes	Yes	Yes	Yes	Yes	NO	Yes
6	NO	Yes	NO	Yes	Yes	Yes	Yes	Yes	Yes	Yes	NO	Yes
7	NO	NO	NO	NO	NO	NO	NO	NO	Yes	NO	NO	NO
8	(yes)	Yes	Yes	(yes)	(yes)	Yes	(yes)	Yes	Yes	(yes)	Yes	Yes
9	Yes	Yes	Yes	Yes	Yes	Yes	Yes	Yes	Yes	Yes	NO	Yes
10	Yes	NO	NO	NO	NO	NO	NO	NO	NO	NO	NO	NO
11	NO	Yes	Yes	Yes	Yes	NO	NO	NO	Yes	Yes	Yes	NO
12	NO	Yes	NO	NO	NO	NO	Yes	Yes	Yes	NO	NO	Yes
13	NO	NO	NO	NO	NO	NO	NO	Yes	Yes	NO	NO	Yes
14	NO	NO	Yes	NO	Yes	NO	NO	NO	Yes	NO	NO	NO
15	NO	NO	Yes	Yes	Yes	Yes	Yes	Yes	NO	Yes	NO	Yes
16	Yes	NO	Yes	Yes	Yes	Yes	Yes	Yes	Yes	Yes	Yes	Yes

*Abbreviations*: *(yes)* partial yes

**Table 11:** AMSTAR 2 assessment: Kappa after first results

		MBO	MBO	MBO		P (Yes)	0.35	(118/192) × (106/192)
		Yes	(yes)	NO		P ((yes))	0.01	(14/192) × (22/192)
FP	Yes	96	8	14	118	P (NO)	0.10	(60/192) × (64/192)
FP	(yes)	0	14	0	14	P (expected)	0.46	0.35 + 0.01 + 0.10
FP	NO	10	0	50	60	P (observed)	0.83	(96 + 14 + 50)/192
		106	22	64	192	Kappa	0.69	(0.83–0.46)/(1–0.46)

*Abbreviations*: *(yes)* partial yes, *Kappa *= (P (observed) – P (expected)) / (1 – P (expected)), *P* probability

## Discussion

### Summary of the main findings

We included four RCTs [12–15] in a meta-analysis on overall survival and on reintervention after EVAR vs OSR of intact AAA, and we used the hazard ratio as the effect measure. Two RCTs [12, 13] did not report the corresponding data on overall survival, and three RCTs [12, 13, 15] did not report the respective data on reintervention. To account for and compensate for the missing data, we deduced the primary data from the pertinent Kaplan-Meier curves. In responding to our request, the author of one RCT [12] conducted an additional survival analysis to separate mortality data from combined data. Regarding the beneficial primary outcome of overall survival, the summary estimate of effect favors neither EVAR nor OSR, HR = 1.02 [95% CI 0.93 to 1.12]. On the other hand, regarding the adverse primary outcome of reintervention, the summary estimate of effect clearly favors OSR, HR = 2.14 [95% CI 1.70 to 2.70]. The risk of bias was judged not to have a noteworthy impact on the results. There is low statistical heterogeneity, *I*^2^ = 0% regarding overall survival and *I*^2^ = 6% regarding reintervention. The funnel plot regarding overall survival is consistent with a low probability of publication bias, though it should be noted that the analysis is based on only four studies. The overview of the study characteristics did not suggest significant clinical heterogeneity. Three RCTs [13–15] had a median follow-up between 9.4 years and 12.4 years which was based on continuous update publications, but a single RCT [12] had a somewhat shorter median follow-up of 3 years which was caused by discontinued funding. We believe that it is not reasonable to exclude this study from the analysis.

### Overall completeness and applicability of evidence

We believe that we have identified the relevant randomized trial studies sufficiently through searching CENTRAL, MEDLINE, and ClinicalTrials.gov. CENTRAL is the result of actively screening for information on RCTs in various literature databases [39]. According to Egger 2003, quote: “investigators should consider the type of literature search and the degree of comprehensiveness that are appropriate for the review in question.” [40]. According to Peinemann 2008, of 11 published RCTs on negative pressure wound therapy, all 11 were obtained from CENTRAL, 10 from MEDLINE, 9 from Embase, and 3 from CINAHL [41]. According to AMSTAR 2, quote: “At least two bibliographic databases should be searched.” [28]. All four included studies were published between 2011 and 2019, and we presume that the principal treatment procedures may not deviate considerably from the current practice. The studies were conducted by public institutions, and a financial conflict of interest was not obvious. The studies provided information required by the Consolidated Standards of Reporting Trials (CONSORT) statement [42]. All included studies have an explanatory attitude. In general, this means enrollment of highly selected participants in an ideal setting in contrast to everyday medical practice [43]. Statistical heterogeneity with respect to the two primary outcomes was low and there was no obvious indication that there was considerable clinical heterogeneity among all four studies. The proportion of patients who did not undergo their assigned intervention was not larger than 10%, and the proportion of patients lost to follow-up was not larger than 5%. Nevertheless, considering the low number of included studies, we believe that the results should be interpreted with caution, and the applicability of the results might be narrow. Since the completion of the four RCTs, stent graft designs and imaging techniques may have been improved, and the described techniques may differ from the actual medical knowledge [44]. Thus, the applicability of the results is presumably limited.

### Limitations and potential biases in the review process

The present review is based on a comprehensive search, and we provide detailed study characteristics and interpretations. The robustness of the findings was supported by sensitivity analyses. The use of the fixed effects model instead of the random effects model did not show a noteworthy impact. Since we did not judge a high risk of bias for any study, we did not compare study data according to the risk of bias. Blinding of participants and personnel (performance bias) and blinding of outcome assessment (detection bias) might be difficult with surgical treatment. We chose to judge an unclear risk in cases when blinding was not reported, and we did the same when blinding was reportedly not applied. We did not create a specific search for SRs, but we are confident that the original search and the application of additional PubMed tools detected all recently published SRs. Both the risk of bias assessment of RCTs and the critical appraisal of SRs are subjective tools. We tried to limit the imbalance of opinion by conducting two independent judgement rounds and clearing the differences by discussion. Our summary estimate of overall survival is in line with all SRs that included the complete follow-up time in calculating the pooled estimate of treatment effect. The application of different effect measures among the SRs, such as odds ratios and hazard ratios, and the inclusion of different data types, such as randomized and nonrandomized data, may serve as an inherent sensitivity analysis corroborating the finding that neither EVAR nor OSR is superior. While checking the SRs, we identified some examples of methodological issues, but the list of potential flaws may not be exhaustive. Nevertheless, they may serve useful hints for future SRs in addition to the advice implied by AMSTAR 2.

We believe that the handling of survival data presents a strength. First, without deducing the data from the Kaplan-Meier curves, the hazard ratios and the respective confidence limits would remain unknown and would introduce a substantial missing data bias. Second, the outcomes table shows both the deduced and, if available, the extracted data. The deduced and the extracted values of the same survival analysis are virtually equivalent. The controlled process of this work introduces certainty and prevents bias. We appreciate that survival curves in the respective papers may converge. Nonetheless, there is no crossing of survival curves concerning the outcomes overall survival and reintervention [45]. We believe that the validity of using the hazard ratios as the primary effect measure is not undermined [46].

### Summary of the main findings of the appraisal of systematic reviews

We thoroughly appraised the methodological quality of 12 published SRs which had evaluated RCTs on EVAR versus OSR [16–27]. It was surprising that six of those SRs [16, 21, 22, 25–27] included nonrandomized studies (NRS) and combined the respective data of RCTs and NRS in the same meta-analysis. It was also surprising that four of the systematic reviews favored odds ratios over risk ratios and that they reported not hazard ratios [16, 19, 22, 24]. Three SRs reported statistical heterogeneity *I*^2^ figures of 76%, 87%, and 89% without considering waiving the summary estimate. 11 meta-analyses resulted in estimates of overall survival that favor neither EVAR nor OSR. The estimate of one meta-analysis [26] appeared to favor OSR, but the analysis contained selected data. Visual inspection of the Kaplan-Meier curves clearly suggested that the proportional hazards assumption was violated. Hence, the authors conducted a split time-point analysis and analyzed only data of the late phase between 1.8 and 5.0 years, whereas data of the first early phase between 0 and 1.8 years were excluded. All SRs except the Cochrane Review by Paravastu [24] had considerable methodological issues that questioned the reliability of their evidence synthesis. Examples are as follows: violation of the proportional hazards assumption; lumping together randomized and nonrandomized data within the same meta-analysis; combining subtotals and thereby producing duplicate data; exclusion of study data based on medium follow-up time; arbitrary exclusion of data based on age; an implausible hazard ratio of greater than 200 of a single observational study questions the credibility of the meta-analysis in question; conducting meta-analyses that display very high statistical heterogeneity; use of odds ratio although effect measures of higher level such as hazard ratio or risk ratio were possible; using split time-point analysis and reporting only the late phase results but not the early phase results. We think that both the present systematic review and the Cochrane Review are comparable in reaching a high methodological standard. Main differences are the interval of more than 10 years between search dates and the use of odds ratio by Paravastu [24] versus hazard ratio by us as the principal overall mortality measure of effect. It has been suggested that odds ratios (odds: events/events) can mislead and should be used only in case-control studies and logistic regression analyses [36]. Stronger designs than case-control studies allow the calculation of risk ratios (risk: events/population) [35]. Thus, according to Deeks 1998, quote: "there is no reason for compromising interpretation by reporting results in terms of odds rather than risks.” Time-to-event data give a more sensitive assessment than dichotomous data because they are “looking not only at how many patients had an event, but also at how long after treatment the event occurred.” [47]. According to Higgins 2024 [48], quote: “The most appropriate way of summarizing time-to-event data is to use methods of survival analysis and express the intervention effect as a hazard ratio.” The hazard can be described as “the chance that at any given moment, the event will occur, given that it hasn't already done so.” [47].

Completion which applies to all four RCTs.

## Conclusions

Pooled data from four randomized controlled trials on intact aortic abdominal aneurysm favor not EVAR regarding overall survival but favor OSR with respect to reintervention.

## Supplementary Information

Additional file 1: PRISMA checklist.

Additional file 2: Table S1.

Additional file 3: Table S2.

## Data Availability

Included in the submitted manuscript.
