# Supplementary material for: Endovascular versus open surgery repair of intact abdominal aortic aneurysm: systematic review of randomized controlled trials and critical appraisal of meta-analyses
Source: Syst Rev. 2026 Feb 20;15:79. doi: 10.1186/s13643-025-03044-2 (PMC12983872; doi:10.1186/s13643-025-03044-2)
Supplement: Supplementary file 2 — Additional file 2: Table S1. [file 13643_2025_3044_MOESM2_ESM.docx]

**S1 Table. Reasons for excluded references**

| **26 references that were read in full-text form but excluded from the review** | **Exclusion reasons** |
| --- | --- |
| ***1 Not population of interest*** |  |
| Luebke T, Brunkwall J. Risk-Adjusted Meta-analysis of 30-Day Mortality of Endovascular Versus Open Repair for Ruptured Abdominal Aortic Aneurysms. Ann Vasc Surg. 2015;29(4):845-63. | Not intact aneurysm |
| ***2 Not outcome of interest*** |  |
| Chen Z, Wang S, Tang XB, Wu ZM, Kou L, Liu H, et al. [Comparison of open and endovascular repair for abdominal aortic aneurysm mid-term outcomes: a single center randomized controlled trial]. Zhonghua Wai Ke Za Zhi. 2011;49(10):869-872. Chinese. | Not overall survival; Chinese language |
| Cuypers PW, Gardien M, Buth J, Peels CH, Charbon JA, Hop WC. Randomized study comparing cardiac response in endovascular and open abdominal aortic aneurysm repair. Br J Surg. 2001;88(8):1059-65. | Not overall survival, cardiac responses |
| ***6 Not study design of interest*** |  |
| Bizos A, Hostalrich A, Chaufour X, Desgranges P, Layese R, Cochennec F, Canoui-Poitrine F. Comparison of Fenestrated Stentgrafts and Open Repair for Juxtarenal Aortic Aneurysms Using a Propensity Score Matching. Ann Vasc Surg. 2023;95:50-61. | Not randomized |
| Chan YC, Morales JP, Gulamhuseinwala N, Sabharwal T, Carmichael M, Thomas S, et al. Large infra-renal abdominal aortic aneurysms: endovascular vs. open repair--single centre experience. Int J Clin Pract. 2007;61(3):373-378. | Not randomized |
| Charbonneau P, Hongku K, Herman CR, Habib M, Girsowicz E, Doonan RJ, et al. Long-term survival after endovascular and open repair in patients with anatomy outside instructions for use criteria for endovascular aneurysm repair. J Vasc Surg. 2019;70(6):1823-1830. | Not randomized |
| Huang Y, Gloviczki P, Oderich GS, Duncan AA, Kalra M, Fleming MD, et al. Outcome after open and endovascular repairs of abdominal aortic aneurysms in matched cohorts using propensity score modeling. J Vasc Surg. 2015;62(2):304-11.e2. | Not randomized |
| Jetty P, Hebert P, van Walraven C. Long-term outcomes and resource utilization of endovascular versus open repair of abdominal aortic aneurysms in Ontario. J Vasc Surg. 2010;51(3):577-83, 83.e1-3. | Not randomized |
| Malas M, Arhuidese I, Qazi U, Black J, Perler B, Freischlag JA. Perioperative mortality following repair of abdominal aortic aneurysms: application of a randomized clinical trial to real-world practice using a validated nationwide data set. JAMA Surg. 2014;149(12):1260-1265. | Not randomized |
| ***17 Not systematic review of interest*** |  |
| Antoniou GA, Juszczak MT, Antoniou SA, Katsargyris A, Haulon S. Editor's Choice - Fenestrated or branched endovascular versus open repair for complex aortic aneurysms: meta-analysis of time to event propensity score-matched data. Eur J Vasc Endovasc Surg. 2021;61(2):228-237. | Propensity score-matched data |
| Becquemin JP, Haupert S, Issam F, Dubar A, Martelloni Y, Jousset Y, et al. Five-year patient outcomes of endovascular abdominal aortic aneurysm repair in the ENDURANT France registry. Eur J Vasc Endovasc Surg. 2021;61(1):98-105. | French database |
| Columbo JA, Stone DH. Using registry data to fill blind spots from randomized trials of abdominal aortic aneurysm repair. JAMA Netw Open. 2022;5(5):e2212092. | Comment on Yei 2022 |
| Deery SE, Schermerhorn ML. Open versus endovascular abdominal aortic aneurysm repair in Medicare beneficiaries. Surgery. 2017;162(4):721-31. | Medicare-matched database; updated by Yei 2022 |
| Doonan RJ, Girsowicz E, Dubois L, Gill HL. A systematic review and meta-analysis of endovascular juxtarenal aortic aneurysm repair demonstrates lower perioperative mortality compared with open repair. J Vasc Surg. 2019;70(6):2054-2064.e3. | RCTs not included |
| Kayssi A, DeBord Smith A, Roche-Nagle G, Nguyen LL. Health-related quality-of-life outcomes after open versus endovascular abdominal aortic aneurysm repair. J Vasc Surg. 2015;62(2):491-498. | Only data on health-related quality of life |
| Kontopodis N, Antoniou SA, Georgakarakos E, Ioannou CV. Endovascular vs open aneurysm repair in the young: systematic review and meta-analysis. J Endovasc Ther. 2015;22(6):897-904. | Updated by Kontopodis 2023 |
| Kontopodis N, Galanakis N, Charalambous S, Matsagkas M, Giannoukas AD, Tsetis D, et al. Editor's Choice - Endovascular aneurysm repair in high-risk patients: a systematic review and meta-analysis. Eur J Vasc Endovasc Surg. 2022;64(5):461-474. | RCTs not included |
| Powell JT, Sweeting MJ, Ulug P, Blankensteijn JD, Lederle FA, Becquemin JP, et al. Meta-analysis of individual-patient data from EVAR-1, DREAM, OVER and ACE trials comparing outcomes of endovascular or open repair for abdominal aortic aneurysm over 5 years. Br J Surg. 2017;104(3):166-178. | Individual-patient data |
| Rittoo D. Randomized study comparing cardiac response in endovascular and open abdominal aortic aneurysm repair. Br J Surg. 2002;89(3):370; author reply | Comment |
| Schermerhorn ML, Buck DB, O'Malley AJ, Curran T, McCallum JC, Darling J, et al. Long-term outcomes of abdominal aortic aneurysm in the Medicare population. N Engl J Med. 2015;373(4):328-338. | Medicare-matched database; updated by Yei 2022 |
| Siracuse JJ, Schermerhorn ML, Meltzer AJ, Eslami MH, Kalish JA, Rybin D, et al. Comparison of outcomes after endovascular and open repair of abdominal aortic aneurysms in low-risk patients. Br J Surg. 2016;103(8):989-994. | New England database |
| Takagi H, Umemoto T. A meta-analysis pooling survival curves in randomized controlled trials and propensity-score matched studies of endovascular versus open abdominal aortic aneurysm repair. Int J Cardiol. 2014;174(3):785-788. | Propensity score-matched data |
| Velickovic VM, Carradice D, Boyle JR, Hamady M, Cleveland T, Neequaye S, et al. Umbrella review and meta-analysis of reconstructed individual patient data of mortality following conventional endovascular and open surgical repair of infrarenal abdominal aortic aneurysm. Expert Rev Cardiovasc Ther. 2023;21(5):347-356. | Umbrella review; individual-patient data |
| Wang G, Sun Y, Lin Z, Fei X. Elective endovascular vs open repair for elective abdominal aortic aneurysm in patients ≥80 years of age: a systematic review and meta-analysis. Vasc Endovascular Surg. 2023;57(4):386-401. | RCTs not included |
| Williams CR, Brooke BS. Effectiveness of open versus endovascular abdominal aortic aneurysm repair in population settings: a systematic review of statewide databases. Surgery. 2017;162(4):707-720. | Statewide inpatient databases |
| Yei K, Mathlouthi A, Naazie I, Elsayed N, Clary B, Malas M. Long-term outcomes associated with open vs endovascular abdominal aortic aneurysm repair in a Medicare-matched database. JAMA Netw Open. 2022;5(5):e2212081. | Medicare-matched database |
