# Supplementary material for: Endovascular versus open surgery repair of intact abdominal aortic aneurysm: systematic review of randomized controlled trials and critical appraisal of meta-analyses
Source: Syst Rev. 2026 Feb 20;15:79. doi: 10.1186/s13643-025-03044-2 (PMC12983872; doi:10.1186/s13643-025-03044-2)
Supplement: Supplementary file 3 — Additional file 3: Table S2. [file 13643_2025_3044_MOESM3_ESM.docx]

**S2 Table. Included references**

| **33 references associated with the four included RCTs and references of the 12 included meta-analyses** | **Study ID** |
| --- | --- |
| ***4 RCTs (21 references)*** |  |
| Becquemin JP. The ACE trial: a randomized comparison of open versus endovascular repair in good risk patients with abdominal aortic aneurysm. J Vasc Surg. 2009;50(1):222-224. | ACE |
| Becquemin JP, Pillet JC, Lescalie F, Sapoval M, Goueffic Y, Lermusiaux P, et al. A randomized controlled trial of endovascular aneurysm repair versus open surgery for abdominal aortic aneurysms in low- to moderate-risk patients. J Vasc Surg. 2011;53(5):1167-1173. | ACE |
| Blankensteijn JD, de Jong SE, Prinssen M, van der Ham AC, Buth J, van Sterkenburg SM, et al. Two-year outcomes after conventional or endovascular repair of abdominal aortic aneurysms. N Engl J Med. 2005;352(23):2398-2405. | DREAM |
| De Bruin JL, Baas AF, Buth J, Prinssen M, Verhoeven EL, Cuypers PW, et al. Long-term outcome of open or endovascular repair of abdominal aortic aneurysm. N Engl J Med. 2010;362(20):1881-1889. | DREAM |
| de Bruin JL, Vervloet MG, Buimer MG, Baas AF, Prinssen M, Blankensteijn JD. Renal function 5 years after open and endovascular aortic aneurysm repair from a randomized trial. Br J Surg. 2013;100(11):1465-1470. | DREAM |
| Prinssen M, Buskens E, Nolthenius RP, van Sterkenburg SM, Teijink JA, Blankensteijn JD. Sexual dysfunction after conventional and endovascular AAA repair: results of the DREAM trial. J Endovasc Ther. 2004;11(6):613-620. | DREAM |
| Prinssen M, Verhoeven EL, Buth J, Cuypers PW, van Sambeek MR, Balm R, et al. A randomized trial comparing conventional and endovascular repair of abdominal aortic aneurysms. N Engl J Med. 2004;351(16):1607-1618. | DREAM |
| van Schaik TG, Yeung KK, Verhagen HJ, de Bruin JL, van Sambeek MRHM, Balm R, et al. Long-term survival and secondary procedures after open or endovascular repair of abdominal aortic aneurysms. J Vasc Surg. 2017;66(5):1379-1389. | DREAM |
| Endovascular aneurysm repair versus open repair in patients with abdominal aortic aneurysm (EVAR trial 1): randomised controlled trial. | EVAR1 |
| Comparison of endovascular aneurysm repair with open repair in patients with abdominal aortic aneurysm (EVAR trial 1), 30-day operative mortality results: randomised controlled trial. | EVAR1 |
| Endovascular aneurysm repair versus open repair in patients with abdominal aortic aneurysm (EVAR trial 1): randomised controlled trial. Lancet. 2005;365(9478):2179-2186. | EVAR1 |
| Brown LC, Epstein D, Manca A, Beard JD, Powell JT, Greenhalgh RM. The UK Endovascular Aneurysm Repair (EVAR) trials: design, methodology and progress. Eur J Vasc Endovasc Surg. 2004;27(4):372-381. | EVAR1 |
| Brown LC, Powell JT, Thompson SG, Epstein DM, Sculpher MJ, Greenhalgh RM. The UK EndoVascular Aneurysm Repair (EVAR) trials: randomised trials of EVAR versus standard therapy. Health Technol Assess. 2012;16(9):1-218. | EVAR1 |
| Brown LC, Thompson SG, Greenhalgh RM, Powell JT. Incidence of cardiovascular events and death after open or endovascular repair of abdominal aortic aneurysm in the randomized EVAR trial 1. Br J Surg. 2011;98(7):935-942. | EVAR1 |
| Greenhalgh RM, Brown LC, Kwong GP, Powell JT, Thompson SG. Comparison of endovascular aneurysm repair with open repair in patients with abdominal aortic aneurysm (EVAR trial 1), 30-day operative mortality results: randomised controlled trial. Lancet. 2004;364(9437):843-8. | EVAR1 |
| Greenhalgh RM, Brown LC, Powell JT, Thompson SG, Epstein D, Sculpher MJ. Endovascular versus open repair of abdominal aortic aneurysm. N Engl J Med. 2010;362(20):1863-71. | EVAR1 |
| Patel R, Sweeting MJ, Powell JT, Greenhalgh RM; EVAR trial investigators. Endovascular versus open repair of abdominal aortic aneurysm in 15-years' follow-up of the UK endovascular aneurysm repair trial 1 (EVAR trial 1): a randomised controlled trial. Lancet. 2016;388(10058):2366-2374. | EVAR1 |
| Lal BK, Zhou W, Li Z, Kyriakides T, Matsumura J, Lederle FA, et al. Predictors and outcomes of endoleaks in the Veterans Affairs open versus endovascular repair (OVER) trial of abdominal aortic aneurysms. J Vasc Surg. 2015;62(6):1394-1404. | OVER |
| Lederle FA, Freischlag JA, Kyriakides TC, Matsumura JS, Padberg FT, Jr., Kohler TR, et al. Long-term comparison of endovascular and open repair of abdominal aortic aneurysm. N Engl J Med. 2012;367(21):1988-1997. | OVER |
| Lederle FA, Freischlag JA, Kyriakides TC, Padberg FT, Jr., Matsumura JS, Kohler TR, et al. Outcomes following endovascular vs open repair of abdominal aortic aneurysm: a randomized trial. Jama. 2009;302(14):1535-1542. | OVER |
| Lederle FA, Kyriakides TC, Stroupe KT, Freischlag JA, Padberg FT Jr, Matsumura JS, et al. Open versus endovascular repair of abdominal aortic aneurysm. N Engl J Med. 2019;380(22):2126-2135. | OVER |
| ***12 meta-analyses*** |  |
| AlOthman O, Bobat S. Comparison of the short and long-term outcomes of endovascular repair and open surgical repair in the treatment of unruptured abdominal aortic aneurysms: meta-analysis and systematic review. Cureus. 2020;12(8):e9683. | AlOthman 2020 |
| Antoniou GA, Antoniou SA, Torella F. Editor's Choice - Endovascular vs. open repair for abdominal aortic aneurysm: systematic review and meta-analysis of updated peri-operative and long-term data of randomised controlled trials. Eur J Vasc Endovasc Surg. 2020;59(3):385-397. | Antoniou 2020 |
| Bulder RMA, Bastiaannet E, Hamming JF, Lindeman JHN. Meta-analysis of long-term survival after elective endovascular or open repair of abdominal aortic aneurysm. Br J Surg. 2019;106(5):523-533. | Bulder 2019 |
| Chen ZG, Tan SP, Diao YP, Wu ZY, Miao YQ, Li YJ. The long-term outcomes of open and endovascular repair for abdominal aortic aneurysm: a meta-analysis. Asian J Surg. 2019;42(10):899-906. | Chen 2019 |
| Giannopoulos S, Kokkinidis DG, Armstrong EJ. Long-term outcomes of endovascular vs open surgical repair for abdominal aortic aneurysms: a meta-analysis of randomized trials. Cardiovasc Revasc Med. 2020;21(10):1253-1259. | Giannopoulos 2020 |
| Kontopodis N, Gavalaki A, Galanakis N, Kantzas M, Ioannou C, Geroulakos G, et al. Systematic review with meta-analysis of endovascular versus open repair of abdominal aortic aneurysm repair in the young. J Endovasc Ther. 2023:15266028231179419. | Kontopodis 2023 |
| Li B, Khan S, Salata K, Hussain MA, de Mestral C, Greco E, et al. A systematic review and meta-analysis of the long-term outcomes of endovascular versus open repair of abdominal aortic aneurysm. J Vasc Surg. 2019;70(3):954-969. | Li 2019 |
| Loufopoulos G, Tasoudis P, Koudounas G, Zoupas I, Madouros N, Sa MP, et al. Long-term outcomes of open versus endovascular treatment for abdominal aortic aneurysm: systematic review and meta-analysis with reconstructed time-to-event data. J Endovasc Ther. 2023:15266028231204805. | Loufopoulos 2023 |
| Paravastu SC, Jayarajasingam R, Cottam R, Palfreyman SJ, Michaels JA, Thomas SM. Endovascular repair of abdominal aortic aneurysm. Cochrane Database Syst Rev. 2014;2014(1):Cd004178. | Paravastu 2014 |
| Shi F, He Y, Wang S, Jia F, Ji C, Zhang J, et al. Endovascular and open surgical repair of abdominal aortic aneurysms: a comparative analysis of western and Chinese studies. Rev Cardiovasc Med. 2020;21(1):75-92. | Shi 2020 |
| Takagi H, Ando T, Umemoto T. Worse late-phase survival after elective endovascular than open surgical repair for intact abdominal aortic aneurysm. Int J Cardiol. 2017;236:427-431. | Takagi 2017 |
| Yokoyama Y, Kuno T, Takagi H. Meta-analysis of phase-specific survival after elective endovascular versus surgical repair of abdominal aortic aneurysm from randomized controlled trials and propensity score-matched studies. J Vasc Surg. 2020 Oct;72(4):1464-1472.e6. | Yokoyama 2020 |
